# Effects of supplementation with lysophospholipids on performance, nutrient digestibility, and bacterial communities of beef cattle

**DOI:** 10.3389/fvets.2022.927369

**Published:** 2022-07-22

**Authors:** Meimei Zhang, Haixin Bai, Yufan Zhao, Ruixue Wang, Guanglei Li, Yonggen Zhang, Peixin Jiao

**Affiliations:** College of Animal Science and Technology, Northeast Agricultural University, Harbin, China

**Keywords:** bacterial community, beef cattle, digestibility, growth performance, lysophospholipids

## Abstract

An experiment was conducted to investigate the influences of supplemental lysophospholipids (LPL) on the growth performance, nutrient digestibility, and fecal bacterial profile, and short-chain fatty acids (SCFAs) of beef cattle. Thirty-six Angus beef cattle [565 ± 10.25 kg body weight (BW)] were grouped by BW and age, and randomly allocated to 1 of 3 treatment groups: (1) control (CON, basal diet); (2) LLPL [CON supplemented with 0.5 g/kg LPL, dry matter (DM) basis]; and (3) HLPL (CON supplemented with 0.75 g/kg, DM basis). The Angus cattle were fed a total mixed ration that consisted of 25% roughage and 75% concentrate (dry matter [DM] basis). The results reveal that LPL inclusion linearly increased the average daily gain (*P* = 0.02) and the feed efficiency (ADG/feed intake, *P* = 0.02), while quadratically increasing the final weight (*P* = 0.02) of the beef cattle. Compared with CON, the total tract digestibilities of DM (*P* < 0.01), ether extract (*P* = 0.04) and crude protein (*P* < 0.01) were increased with LPL supplementation. At the phylum-level, the relative abundance of *Firmicutes* (*P* = 0.05) and ratio of *Firmicutes*: *Bacteroidetes* (*P* = 0.04) were linearly increased, while the relative abundances of *Bacteroidetes* (*P* = 0.04) and *Proteobacteria* (*P* < 0.01) were linearly decreased with increasing LPL inclusion. At the genus-level, the relative abundances of *Clostridium* (*P* < 0.01) and *Roseburia* (*P* < 0.01) were quadratically increased, and the relative abundances of *Ruminococcus* was linearly increased (*P* < 0.01) with LPL supplementation. Additionally, increasing the dose of LPL in diets linearly increased the molar proportion of butyrate (*P* < 0.01) and total SCFAs (*P* = 0.01) concentrations. A conclusion was drawn that, as a promising feed additive, LPL promoted growth performance and nutrient digestibility, which may be associated with the change of fecal microbiome and SCFAs.

## Introduction

Lipids, which mainly include fats and oils, are commonly added to livestock diets as a concentrated energy source, providing fat-soluble vitamins and essential fatty acids (FAs), promoting the absorption of fat-soluble nutrients and being significant factors in biochemistry, physiology, and nutrition (1, 2). The use of fats as a partial substitute for grains in diets can effectively improve the energy concentration and fattening performance of beef cattle, which is a crucial method for producing marble beef and improving the carcass quality of cattle (3). However, the use of excessive fat (6–7% of the dietary dry matter [DM]) in the ruminant diet can lead to excess visceral fat accumulation and loss of vitamins A and E, furthermore decreasing the growth performance and digestibility of beef cattle (4, 5). As suggested by Souza et al. (6), the digestibility of fatty acids will be decreased with the increase of fatty acid flow to the intestine and the insufficient secretion of intestinal bile salts and pancreatic lipase. In previous studies, it has been found that emulsifiers can compensate for the insufficient of bile secretion and promote the incorporation of fatty acids into micelles, so as to improve fat digestibility (7).

Lysophospholipids (LPL), as a promising feed additive, have been widely used in the diets of non-ruminant animals to improve production and feed efficiency. According to studies by Zhao et al. (8) and Brautigan et al. (9), increases in growth performance, feed efficiency, and dietary nutrient absorption were observed when diets supplemented with LPL were fed to pigs and poultry. Studies have shown that LPL increased dietary fat absorption due to the emulsification property thereof and upregulated of the expression of various genes such as GAS6 and RAMP2 in the intestinal epithelium (9). Despite such findings, there is a scarcity of research on the effect of dietary LPL inclusion in ruminants.

Gut microbiota, serving as an important barrier in the host, has been known to influence the health of animals. Gut microbiota is significant factor in nutritional, metabolic and immunological functions, while also having effects on the growth performances and feed efficiency of livestock animals (10). The composition of gut microbiota is strongly influenced by diet, animal age, host genetics and feed additive (11, 12). Jang et al. (13) reported that supplementation of LPL in lactation diets increased the *Firmicutes* to *Bacteroidetes* ratio and improved intestinal health. Moreover, Qiu et al. (14) demonstrated that the supplementation of choline, as one of the main components of LPL, can enhance the growth performance and gut health of weaned piglets by altering the gut microbiota and metabolites. Therefore, further clarifying the effects of LPL inclusion in the rations of beef cattle on gut microbiome is of considerable significance. We hypothesized that adding LPL would improve growth performance, feed efficiency and nutrient digestibility due to regulating gut microbiota, and promoting the gut health of beef cattle. Thus, the objective of the present study was to investigate the effect of supplementing diets with LPL on the performance, apparent digestibility, fecal bacterial communities, and SCFAs of beef cattle.

## Materials and methods

All experimental procedures involving animals in the present study were approved by the Institutional Animal Care Committee (Protocol number: NEAU- [2011]-9), Northeast Agricultural University (Harbin, China).

### Lysophospholipids products

The LPL product used in the present experiment was a hydrolyzed soy lecithin, which is composed of phospholipids, free fatty acids and LPL (30%), and was provided by Jianming (China) Technology Co., LTD (Zhuhai, China).

### Animals, experimental design, and diets

Thirty-six Angus beef cattle were blocked for body weight (BW) (565 ± 10.3 kg) and randomly assigned to 1 of 3 treatments (12 beef cattle per treatment): (1) control (CON; basal diet); (2) LLPL [CON supplemented with low LPL, 0.5 g/kg of dry matter (DM) basis]; and (3) HLPL (CON supplemented with high LPL, 0.75 g/kg of DM basis). All the cattle were housed in individual pens (4 × 3 m^2^) with free access to water and were fed *ad libitum* twice daily at 800 and 1,700 h. The cattle were fed for 74 days. During the first 14 days, the feed intake of concentrates was gradually increased, until the ratio of concentrate to roughage in the final diet (the first 10 days) reached 75:25, following which the feed intake was gradually increased until reaching the arbitrary feed intake. The dietary ingredients and chemical composition are presented in Table 1. The Chinese wild rye grass (*Leymus chinensis*) which is widely distributed throughout the Eurasian Steppe, including the Songnen Plain and the eastern Inner Mongolian Plateau in China, is a perennial species of Gramineae.

**Table 1:** Ingredients and chemical composition of the experimental diet (DM basis).

	**Diet** ^a^		
**Item**	**CON**	**LLPL**	**HLPL**
**Ingredient composition, % of DM**			
Corn grain	46	46	46
Soybean meal	5	5	5
Peanut hull	10	10	10
Soybean hull	5	5	5
Chinese wild rye grass	10	10	10
Distillers dried grains with soluble	12	12	12
Calcium bicarbonate	0.5	0.5	0.5
Corn germ meal	5	5	5
Rumen bypass fat	2.5	2.5	2.5
Molasses	0.5	0.5	0.5
Salt1	0.7	0.7	0.7
Limestone	1.1	1.05	1.03
Magnesium oxide	0.3	0.3	0.3
Sodium bicarbonate	1	1	1
Mineral-vitamin premix^b^	0.4	0.4	0.4
Lysophospholipids	0	0.050	0.075
**Chemical composition, % of DM**			
OM	92.3	92.2	92.4
CP	11.6	11.6	11.7
DM	88.6	89.1	88.5
EE	6.8	6.8	6.9
NDF	26.5	26.6	26.6
ADF	15.8	15.8	15.6

a *CON, control; LLPL, 0.5 g/kg lysophospholipids; HLPL, 0.75 g/kg lysophospholipids*.

b *The Mineral-vitamin premix provided the following per kilogram of the diet: VA 6000 IU, VD 600 IU, VE 50 IU, Fe 10 mg, Cu 15.0 mg, Mn 27 mg, Zn 65 mg, I 0.50 mg, Co 0.20 mg*.

### Sample collection

The feed offered and refused for individual beef cattle was weighed every day throughout the trial to calculate DM intake (DMI). To determine the average daily gain (ADG), all the cattle were weighed at the end of the diet adaptation period (d 15), at the midpoint on 2 consecutive days (d 29 and 30), and at the end of the experiment on two consecutive days (d 59 and 60). Samples of dietary ingredients, diets, and refusals during the experimental period were collected and stored at −20°C, and later pooled by beef cattle and period. Then, all the feed samples were dried at 55°C for 48 h, ground to pass through a 1-mm screen, and stored for chemical analysis.

Fecal samples (about 500 g fresh) were collected from the rectum of individual animal daily at 600, 1,200, 1,800, and 2,400 h from d 28 to 30, and from d 58 to 60 according to the method of Yang et al. (15). The collected fecal samples were pooled by animal and by sampling period, dried at 55°C for 48 h, and ground to pass through a 1-mm screen for subsequent chemical analysis. Meanwhile, ~5 g spot fecal samples were collected, and then stored instantly in liquid nitrogen until determination of bacterial communities and SCFAs.

### Chemical analysis

Samples of individual feed ingredients and refusals were dried at 55°C for 48 h for DM (934.01), crude protein (CP; 976.05), ether extract (EE; 920.39), and ash (942.05) determination (16). Following the procedure described by Van Soest et al. (17), the neutral detergent fiber (NDF) content was analyzed using heat-stable α-amylase and sodium sulfite. The acid detergent fiber (ADF) content was analyzed according to AOAC (1990; method 954.01). The total-tract apparent nutrient digestibility was determined using acid detergent insoluble ash as an internal marker. The acid detergent insoluble ash contents of feeds and feces were determined by analyzing samples for ADF, as previously described, and the ash contents were determined by incinerating the Ankom bags for 8 h at 450°C in a muffle furnace (18). The DM, EE, CP, NDF, and ADF were analyzed using similar methods as described for the analyses of feed and fecal samples. The apparent digestibility was measured according to the method described by Merchen (19).

The fecal samples for determining microbial profiles were delivered to Personalbio Biotechnology Co., Ltd. (Shanghai, China) for 16S rRNA high-throughput Sequencing according to the method described by Zhu et al. (20). In brief, OMEGA Soil DNA Kits (M5635-02) (Omega Bio-Tek, Norcross, GA, USA) were used for DNA extraction of fecal samples according to the manufacturer's instructions. The quantity and quality of extracted DNA were determined by NanoDrop NC2000 spectrophotometer and agarose gel electrophoresis, and stored at −80°C until further processing. The V3–V4 region of the bacteria 16S rRNA gene was amplified by polymerase chain reaction (PCR) (1 cycle 95°C for 5 min, followed by 25 cycles at 95°C for 30 s, 50°C for 30 s and 72°C of 40 s, and a final extension of 72°C for 7 min). The amplification was performed using the forward primer 338F (5′-ACTCCTACGGGAGGCAGCA-3′) and the reverse primer 806R (5′-5′-GGACTACHVGGGTWTCTAAT-3′). The amplified product concentration according to PCR was mixed with equal concentration. Amplicons were purified by 2% agarose gels and recycled by using the AxyPrep DNA Gel Extraction Kit (Axygen Biosciences, Union City, CA, USA) according to the manufacturer's instructions, being for being quantified by means of the Quant-iT PicoGreen dsDNA Assay Kit.

The concentrations of SCFAs were determined in feces using gas chromatography (GC-2010, Shimadzu Corporation, Kyoto, Japan) according to the described method by Zhao et al. (21). In brief, the fecal samples (200 mg) were homogenized in 2 ml of ultrapure water by Vortex-Genie for 2 min. The mixture was centrifuged at 10,000 rpm for 10 min at 4°C, then the supernatant was filtered through a 0.22 μm filter membrane, which was repeated three times. The filtered supernatant was mixed with 25% (W/V) metaphosphoric acid at a ratio of 5:1 for the SCFAs assay.

### Statistical analyses

All data were analyzed using the Proc Mixed procedure of SAS (version 9.2; SAS Institute Inc., Cary, NC). The statistical model included week, treatment, and interaction of treatment × week as fixed effects, as well as beef cattle within treatment as a random effect. The significance among treatments was analyzed using Tukey's multiple comparison test. To analyze the linear and quadratic effects of LPL inclusion levels, a polynomial orthogonal contrast was conducted. Significant differences were declared at *P* ≤ 0.05 and trends at 0.05 < *P* ≤ 0.10.

## Results

### Growth performance

As shown in Table 2, supplementation of LPL in the diets did not affect the DMI. However, increasing the supplemental dose of LPL linearly increased the ADG (*P* = 0.02) and feed efficiency (*P* = 0.02), and quadratically increased the final BW (*P* = 0.02). In comparison with CON, supplementation of LPL tended to increase the ADG (*P* = 0.07) and the feed efficiency (*P* = 0.06).

**Table 2:** Effect of dietary lysophospholipids supplementation on growth performance of beef cattle.

	**Treatment^B^**				***P*** **-value** ^D^		
**Item** ^A^	**CON**	**LLPL**	**HLPL**	**SEM** ^C^	**Treatment**	**Linear**	**Quadratic**
Initial weight, kg	565	562	566	15.221	0.67	0.98	0.83
Final weight, kg	628	633	653	3.380	0.68	<0.01	0.02
ADG, kg/d	1.36	1.45	1.53	0.077	0.07	0.02	0.68
Feed efficiency	118.1^b^	127.2^ab^	134.7^a^	4.675	0.06	0.02	0.73
DMI, kg/d	11.55	11.42	11.37	0.084	0.2317	0.12	0.94

a, b *Means within a row with different superscripts differ (P <0.05)*.

A *ADG, average daily gain; DMI, dry matter intake; FCR, feed conversion ratio*.

B *CON, control; LLPL, 0.5 g/kg lysophospholipids; HLPL, 0.75 g/kg lysophospholipids*.

C *SEM, standard error of the mean*.

D *Treatment, contrast between CON, LLPL and HLPL; Linear, linear effect of LPL addition; Quadratic, quadratic effect of LPL addition*.

### Nutrient digestibility

The digestibility of DM (*P* < 0.01), EE (*P* = 0.04) and CP (*P* < 0.01) increased linearly with increasing doses of LPL. However, the apparent digestibilities of ADF and NDF showed no significant difference among the treatment groups (Table 3). The digestibilities of DM (*P* < 0.01), EE (*P* = 0.04), and CP (*P* < 0.01) were higher for LLPL and HLPL compared with CON.

**Table 3:** Effect of dietary lysophospholipids supplementation on nutrient digestibility of beef cattle.

	**Treatment^B^**				***P*** **-value** ^D^		
**Item** ^A^	**CON**	**LLPL**	**HLPL**	**SEM** ^C^	**Treatment**	**Linear**	**Quadratic**
**DM**	**63.40^b^**	**67.31^a^**	**67.15^a^**	**0.558**	** <0.01**	** <0.01**	**0.05**
**EE**	**68.32^b^**	**71.82^a^**	**71.28^a^**	**0.741**	**0.04**	**0.04**	**0.21**
**CP**	**60.14^b^**	**64.44^a^**	**65.30^a^**	**0.554**	** <0.01**	** <0.01**	**0.45**
**NDF**	**36.03**	**35.57**	**36.59**	**0.926**	**0.87**	**0.65**	**0.88**
**ADF**	**26.26**	**25.90**	**26.76**	**0.671**	**0.62**	**0.71**	**0.41**

a, b *Means within a row with different superscripts differ (P <0.05)*.

A *DM, dry matter; EE, ether extract; CP, crude protein; NDF, neutral detergent fiber; ADF, acid detergent fiber*.

B *CON, control; LLPL, 0.5 g/kg lysophospholipids; HLPL, 0.75 g/kg lysophospholipids*.

C *SEM, standard error of the mean*.

D *Treatment, contrast between CON, LLPL and HLPL; Linear, linear effect of LPL addition; Quadratic, quadratic effect of LPL addition*.

### Sequence and fecal bacterial community composition

After removing incorrect and chimeric sequences, 728961 sequencing reads were generated from all the 15 samples. Each sample returned 29953~51192 sequences, with an average length of 421 bp (Supplementary Table 1). After ASV picking and chimera checking, 42079 amplicon sequence variants (ASVs) were obtained for all the samples at 100% sequence similarity. In total, 6778, 6445, and 5958 ASVs were clustered in the CON, LLP and HLPL groups, respectively; further, 2194 ASVs were exclusive in all three treatments (Supplementary Figure 1). Similarly, the Good's coverages of all samples exceeded 99%, thereby verifying the accuracy and reproducibility of the sequencing.

A total of 15 bacterial phyla were detected in all the samples. *Firmicutes* (70.28%), *Bacteroidetes* (25.54%), and *Spirochaetes* (1.32%) were the most predominant phyla (Supplementary Table 2). At the genus level, a total of 249 bacterial genera were detected, and the six most dominant genera were successively *unclassified Ruminococcaceae* (23.87%), *unidentified Bacteroidales* (11.64%), *Clostridium* (6.35%), *unidentified Peptostreptococcaceae* (6.62%), *unidentified Peptostreptococcaceae* (4.23%), and 5-7N15 (3.74%) (Supplementary Table 3).

### Fecal bacterial communities

The alpha bacterial diversity is shown in Table 4. The indexes of Chao1, Shannon, Simpson, Observed species and Faith's PD did not differ among treatments. The results of the rarefaction curves indicate that with the increase in sequence number, the number of ASV in feces from each sample showed a trend of increasing first and then becoming stable. As such, the sequencing depth in the present study was sufficient for evaluating dominant members of the fecal bacterial community (Supplementary Figure 2).

**Table 4:** Effect of dietary lysophospholipids supplementation on Alpha diversity index of fecal bacteria.

	**Treatment^A^**				***P*-value^C^**		
**Item**	**CON**	**LLPL**	**HLPL**	**SEM^B^**	**Treatment**	**Linear**	**Quadratic**
Chao1	3694.76	3706.71	3576.77	336.712	0.94	0.83	0.83
Observed species	3082.82	3021.60	2936.52	206.830	0.86	0.64	0.89
Shannon	9.64	9.84	9.60	0.178	0.56	0.96	0.34
Simpson	0.99	0.99	0.99	0.002	0.18	0.70	0.13
Faith's PD	147.16	142.34	135.34	7.087	0.46	0.28	0.73

A *CON, control; LLPL, 0.5 g/kg lysophospholipids; HLPL, 0.75 g/kg lysophospholipids*.

B *SEM, standard error of the mean*.

C *Treatment, contrast between CON, LLPL and HLPL; Linear, linear effect of LPL addition; Quadratic, quadratic effect of LPL addition*.

**Figure 1:**
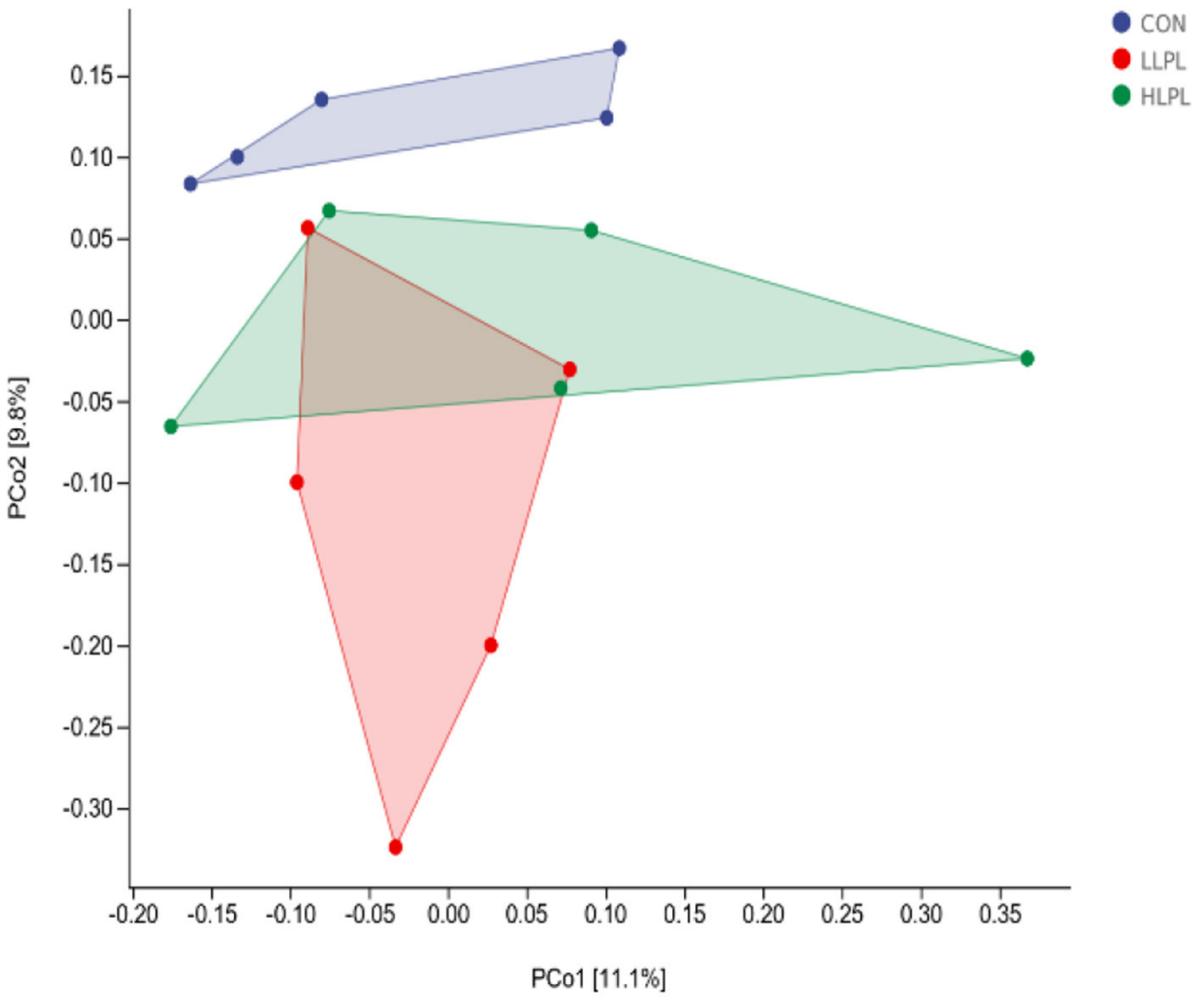
Principal coordinate analysis (PCoA) of the fecal microbial communities based on unweighted UniFrac. CON, control; LLPL, 0.5 g/kg lysophospholipids; HLPL, 0.75 g/kg lysophospholipids.

As revealed by principal coordinates analysis (PCoA) based on unweighted UniFrac metrics, there was a distinct clustering of the microbiota composition between CON and LPL treatments (Figure 1). The results of ANOSIM analysis reveal a significant difference in bacterial community composition between the treatments of CON and LLPL (*R* = 0.350, *P* = 0.029). There was a trend of difference between the treatments of LLPL and HLPL (*R* = 0.244, *P* = 0.099). In addition, there was no significant difference between the CON and HLPL treatments (*R* = 0.096, *P* = 0.206).

**Figure 2:**
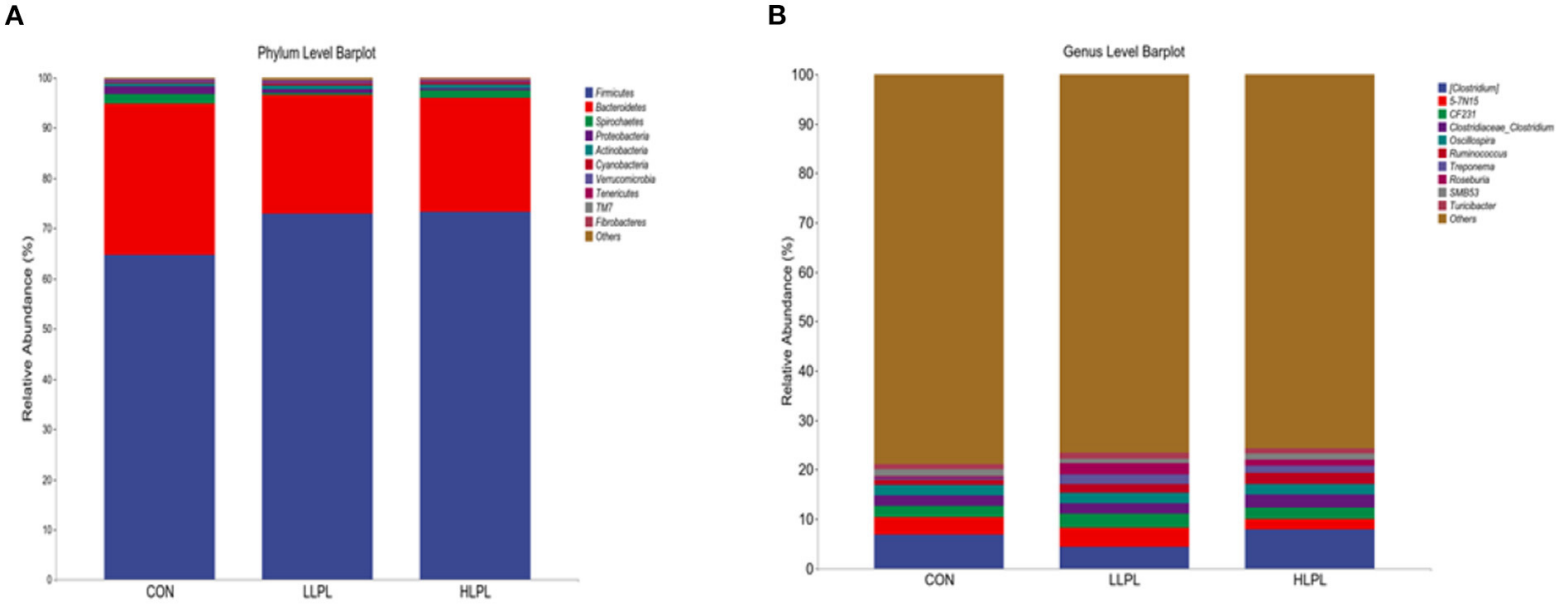
Fecal bacterial phyla and genera in three treatments. **(A)** The bacterial taxonomic composition of fecal samples from the three treatments at the phylum level. **(B)** The bacterial taxonomic composition of fecal samples from the three treatments at the genus level (top 10, according to relative abundance). CON, control; LLPL, 0.5 g/kg lysophospholipids; HLPL, 0.75 g/kg lysophospholipids.

The results of the relative abundances (top 10) of the bacterial phyla and genus in the feces of cattle are shown in Table 5 and Figure 2. At the phylum level, with increases in dietary LPL levels, the relative abundances of *Firmicutes* (*P* = 0.05) linearly increased, and the relative abundances of *Bacteroidetes* (*P* = 0.04) linearly decreased. Thus, increasing the supplementation of LPL linearly increased (*P* < 0.01) the F:B ratio (*Firmicutes*: *Bacteroidetes*). Moreover, with the inclusion of LPL, a linear decrease (*P* < 0.01) in the relative abundance of *Proteobacteria* was observed. In comparison with CON, the F:B ratio was increased (*P* < 0.01), whereas the relative abundances of *Proteobacteria* were decreased (*P* < 0.01) with LPL supplementation. At the genus level, with the increase in supplemental dose of LPL, the relative abundances of *Clostridium* (*P* < 0.01) and *Roseburia* (*P* < 0.01) quadratically increased, and the relative abundances of *Ruminococcus* linearly increased (*P* < 0.01). Moreover, the relative abundances of *Ruminococcus* (*P* = 0.01), *Treponema* (*P* < 0.01), and *Roseburia* (*P* < 0.01) were higher for LLPL and HLPL compared with CON.

**Table 5:** Effects of dietary lysophospholipids on the relative abundance (%) of fecal bacteria.

	**Treatment** ^B^				***P*** **-value** ^D^		
**Item** ^A^	**CON**	**LLPL**	**HLPL**	**SEM** ^C^	**Treatment**	**Linear**	**Quadratic**
**Phylum**
*Firmicutes*	64.68	72.93	73.23	2.897	0.08	0.05	0.51
*Bacteroidetes*	30.09	23.72	22.81	2.343	0.06	0.04	0.61
**F: B^C^**	2.15^b^	3.49^a^	3.28^a^	0.199	<0.01	<0.01	0.04
*Spirochaetes*	2.02	0.37	1.56	0.469	0.08	0.28	0.05
*Proteobacteria*	1.42^a^	0.71^b^	0.33^c^	0.086	<0.01	<0.01	0.87
*Actinobacteria*	0.60	0.66	0.67	0.379	0.98	0.89	0.97
*Cyanobacteria*	0.24	0.38	0.38	0.128	0.60	0.41	0.80
*Verrucomicrobia*	0.27	0.39	0.24	0.107	0.58	0.96	0.33
*Tenericutes*	0.21	0.25	0.22	0.035	0.80	0.82	0.44
*TM7*	0.17	0.15	0.12	0.045	0.76	0.48	0.86
*Fibrobacteres*	0.18	0.11	0.18	0.045	0.66	0.35	0.39
*Others*	0.18	0.29	0.25	0.034	0.21	0.38	0.23
**Genus**							
*Clostridium*	6.82^b^	4.30^c^	7.92^a^	0.396	<0.01	0.42	<0.01
*5-7N15*	3.53	3.97	2.42	0.625	0.18	0.17	0.33
*CF231*	2.27	2.74	2.40	0.754	0.89	0.96	0.66
*Clostridiaceae_Clostridium*	2.02	2.15	2.50	0.509	0.75	0.51	0.96
*Oscillospira*	2.14	2.07	2.23	0.199	0.94	0.69	0.70
*Ruminococcus*	1.05^b^	1.79^a^	2.15^a^	0.172	0.01	<0.01	0.99
*Treponema*	0.36^c^	2.01^a^	1.15^b^	0.132	<0.01	0.06	0.18
*Roseburia*	0.40^c^	2.28^a^	1.16^b^	0.141	<0.01	<0.01	<0.01
*SMB53*	1.45	0.87	1.19	0.285	0.43	0.40	0.28
*Turicibacter*	1.12	1.14	1.08	0.452	0.99	0.97	0.94
*Others*	78.83	76.64	75.71	1.636	0.28	0.20	0.95

a, b, c *Means within a row with different superscripts differ (P <0.05)*.

A *F:B, Firmicutes: Bacteroidetes*.

B *CON, control; LLPL, 0.5 g/kg lysophospholipids; HLPL, 0.75 g/kg lysophospholipids*.

C *SEM, standard error of the mean*.

D *Treatment, contrast between CON, LLPL and HLPL; Linear, linear effect of LPL addition; Quadratic, quadratic effect of LPL addition*.

### Association and model predictive analysis

**Figure 3:**
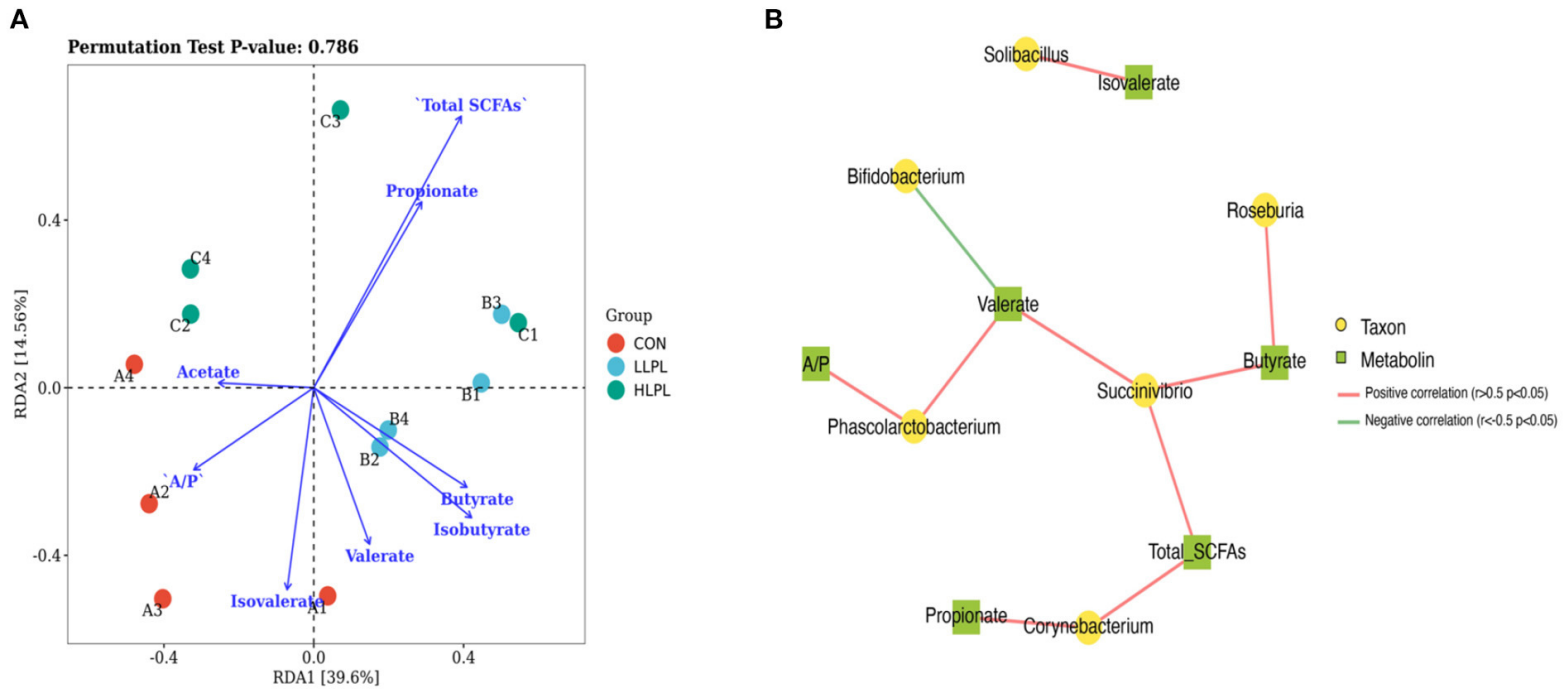
Association and model predictive analysis. **(A)** RDA analysis of the correlation between the microbiota and their metabolites. Each point represents a sample; each arrow represents a metabolite (SCFAs). **(B)** Co-occurrence network analysis among the microbiota and metabolites. Each co-occurring pair among microbial populations at the genus level and their metabolites has an absolute Spearman rank correlation above 0.5 [red line, positive correlation (*r* ≥ 0.5); green line, negative correlation (*r* ≤ −0.5)] with a FDR-corrected significance level under 0.05.

Redundancy analysis (RDA) of the correlation between the microbiota and their metabolites (SCFAs; Figure 3A). The correlation between microbiota distribution and metabolites were as follows: Total SCFAs > Propionate > Isobutyrate > Isovalerate > Butyrate > A/P > Valerate > Acetate. In addition, RDA indicated that there was positive correlation among Total SCFAs, Propionate, and Butyrate, and negative correlation between Total SCFAs and Isobutyrate, Isovalerate, A/P and Acetate. Co-occurrence network analysis was conducted between the top 30 bacterial genera and their metabolites (Figure 3B). The results indicated that *Succinivibrio* is positively correlated with Total SCFAs, Butyrate and valerate, and *Roseburia* is positively Butyrate. *Corynebacterium* is positively correlated with Propionate and Total SCFAs. *Phascolarctobacterium* is positively correlated with A/P and Valerate, however *Bifidobacterium* is negatively correlated with Isovalerate.

### Total VFA concentration and VFA profiles

Increasing the dose of LPL in diets, the molar proportion of butyrate (*P* < 0.01), and total SCFAs (*P* = 0.01) concentrations linearly increased, as shown in Table 6. However, proportions of acetate, propionate, valerate, isobutyrate, and isovalerate, and the ratio of acetate to propionate showed no significant difference among the treatment groups. Compared with CON, the supplementation of LPL resulted in higher total SCFAs concentrations (*P* = 0.02) and molar proportion of butyrate (*P* < 0.01).

**Table 6:** Effect of dietary lysophospholipids supplementation on the fermentation of SCFAs in beef cattle.

	**Treatment** ^B^				***P*** **-value** ^D^		
**Item** ^A^	**CON**	**LLPL**	**HLPL**	**SEM** ^C^	**Treatment**	**Linear**	**Quadratic**
Total SCFAs, mM	39.82^b^	44.11^a^	43.76^a^	0.970	0.02	0.01	0.20
Acetate, mol/100 mol	62.35	61.69	61.19	1.241	0.39	0.37	0.81
Propionate, mol/100 mol	23.32	22.75	22.88	0.582	0.65	0.46	0.75
Butyrate, mol/100 mol	7.93^b^	9.30^a^	9.63^a^	0.267	<0.01	<0.01	0.35
Valerate, mol/100 mol	1.65	1.60	1.62	0.078	0.55	0.54	0.40
Isobutyrate, mol/100 mol	2.88	2.86	2.83	0.154	0.52	0.25	0.46
Isovalerate, mol/100 mol	1.87	1.80	1.85	0.148	0.34	0.44	0.40
A:P ratio	2.71	2.70	2.69	0.137	0.19	0.20	0.61

a, b *Means within a row with different superscripts differ (P <0.05)*.

A *Total SCFAs, total short-chain fatty acids; A:P, acetate: propionate*.

B *CON, control; LLPL, 0.5 g/kg lysophospholipids; HLPL, 0.75 g/kg lysophospholipids*.

C *SEM, standard error of the mean*.

D *Treatment, contrast between CON, LLPL and HLPL; Linear, linear effect of LPL addition; Quadratic, quadratic effect of LPL addition*.

## Discussion

### Growth performance

To the best of our knowledge, this is the first study to evaluate the effect of LPL inclusion on the growth performance of finishing cattle. Previous studies of LPL as feed additives have mostly focused on improving the growth performance and feed efficiency of monogastric animals (22, 23). Consistent with the findings in monogastric animals, increasing the supplemental dose of LPL in diets was found to linearly increase the ADG and decrease the FCR in the present study, indicating an improvement in growth performance. In prior research, phospholipids (a source of LPL) in the rumen were reported to escape microbial degradation and increase emulsification in the small intestine (24, 25). The improved performance of cattle with LPL supplementation may have been due to the increase in the absorption of nutrients in the small intestine in the current study. To the present knowledge, the effects of LPL as a feed additive on growth performance in ruminants have only been examined in 2 studies. Reis et al. (7) conducted a recent experiment and found that supplementation with LPL improved growth performance and feed efficiency without affecting the DMI of dairy cows. However, Song et al. (26) suggested that supplementation with LPL (0.3 or 0.5% w/w) did not affect the growth performance of Hanwoo heifers. The inconsistent results between Song et al. (26) and the present study could be partly attributed to the different types of LPL product, sources of phospholipids and fat, enzymatic (phospholipase) hydrolysis processes to produce LPL, and concentrations of LPL in the product. Notably, LPL has been widely used in the diets of non-ruminants as an additive, and the consistent effects of LPL on animal growth performance have been observed, which indicates that the degree of ruminal bypass of LPL might be critical for the effects of LPL on the growth performance of beef cattle (8, 22, 27).

### Nutrient digestibility

Numerous studies on vitro digestion have shown that dietary emulsifiers can modulate the direct contact of lipid substrates and lipase, and thus, promote lipid digestion (28, 29). LPL could improve the nutrient digestibility of non-ruminant animals, which can be mainly attributed to the emulsification characteristics thereof (23, 30). In the present study, the linearly increased in the digestibilities of DM, EE, and CP with increasing supplementation of LPL, which is consistent with the findings of Song et al. (26) who found that supplementation of dietary LPL (0.3 or 0.5% w/w) increased the DM digestibility of beef cattle. In the present study, an assumption was made that the nutrient digestibility of EE was improved due to LPL being able to effectively reduce the size of fat globules and form smaller micelles in the guts of animals, thereby increasing the larger surface areas of lipid droplets for pancreatic lipases to interact so that more fatty acids would be incorporated (7, 31). In addition, the improved digestibility of CP could be attributed to LPL modifying the lipid bilayer of the membrane and increasing the number and size of the membranous pores, thereby altering the fluidity of the membrane and the transmembrane permeabilities of the nutrients (32, 33). However, LPL supplementation in ruminants does not always have the same effect on nutrient digestibility. Huo et al. (34) reported that supplementation of 0.05% LPL (DM basis) in the diets of lambs increased the digestibilities of DM, CP and OM. However, in feeding 0.05 and 0.075% LPL of the dietary DM to dairy cows, Lee et al. (25) observed a slight decrease in DM digestibility compared with the control group. The inconsistent results between Lee et al. (25) and the present study could be partially attributed to differences in diet, genetics and enzymatic (phospholipase) hydrolysis processes to produce LPL.

### Fecal bacterial community composition

The results of PCoA with the unweighted UniFrac metrics reveal that the samples of the LPL group were clearly separated compared with the samples of the CON, indicating that the microbial composition of cattle in the LPL group was different from that of the CON group. The AMOVA results also show that there were significant differences between LLPL and CON. As revealed by numerous findings, gut microbiota are significant factors in animal health and growth, being responsible for nutrient metabolism, energy utilization and regulation of the immune responses in livestock (35, 36). To the present knowledge, no information is available on gut microbiota affected by dietary emulsifier supplementation in cattle. In the present study, *Firmicutes* and *Bacteroidetes* were the most predominant phyla in the fecal microbial communities, which is consistent with previous studies (37, 38). Additionally, a linear increase in the relative abundance of *Firmicutes* and a linear decrease in the relative abundance of *Bacteroidetes* with increasing supplemental dose of LPL were observed, resulting in an increased ratio of F:B (*Firmicutes*:*Bacteroidetes*), which could be beneficial for weight gain (39). A recent experiment conducted by Jang et al. (13) showed that LPL supplementation in diets increased the F:B ratio in the jejunum of sow, which is consistent with the present study (37). In addition, the increase in *Firmicutes* and decrease in *Bacteroidetes* and *Proteobacteria* in the feces seemed to have a positive impact with LPL on the gut health of cattle. Such positive impact could be attributed to *Firmicutes* mainly including beneficial bacteria and *Proteobacteria* mainly including harmful bacteria (35). The increase in *Proteobacteria* can lead to an imbalance of gut microbes and metabolic disorders (40). Moreover, study has reported that *Bacteroides* in animal fecal matter (41) has been known as fecal indicator bacteria (FIB). Fang et al. (42) found that *Ruminococcus* was positively correlated with the finishing weight, whereas the microbial taxa related to intestinal damage and inflammation showed opposite effects. Consistent with such findings, the abundance of *Ruminococcus* increased in the present study, which could partly explain the increased ADG with LPL supplementation.

### Fecal fermentation characteristics

Short chain fatty acids are the gut microbiota metabolites produced from fermentation of complex carbohydrates, and are significant factors in maintaining intestinal health, regulation of glucose and lipid metabolism and epithelial barrier function (43, 44). In the present study, the increased the total concentration of SCFAs and molar proportion of butyrate in feces with increasing dietary supplementation of LPL is consistent with report by Qiu et al. (14) that choline, as one of the main components of LPL, could increase the concentration of SCFAs and molar proportion of butyrate of weaned piglets. Using RDA analysis, total SCFAs, Propionate, and Butyrate were significantly associated with feces microbiota. Furthermore, Spearman correlation analysis showed that *Roseburia* is positively Butyrate. The beneficial effects of LPL supplementation on SCFAs production were supported by the observation of enrichment of SCFA-producing bacteria, such as *Roseburia* and *Clostridium* (45, 46). Gut microbial butyrate metabolic pathways have been reported to increase energy intake and improve intestinal histology (for example, villi length, and crypt depth) in livestock, thereby having beneficial effects on livestock body weight (37, 47, 48). Moreover, Bedford and Gong (49) reported that butyrate has modulating capacity on energy and metabolism, which has a positive effect on body weight gain in animals. Therefore, the linearly increased ADG and decreased FCR could be a result of the greater proportion of butyrate with supplemented LPL in the diets of beef cattle in the present study.

## Conclusion

Supplementation of beef cattle diets with LPL could promote growth performance, feed efficiency and apparent digestibility, which may be related to the change of relative abundance of bacterial communities, total SCFAs concentration and SCFAs profiles. The findings of the present study provide essential insights into the use of LPL as a growth promoter in beef cattle, and imply that manipulating the gut microbial community could be an efficient strategy for improving the finishing weight in the beef cattle industry. Furthermore, this study provides quantitative information that 0.75 g/kg LPL may be the optimal supplemental level for beef cattle finishing diets.

## Data availability statement

The datasets presented in this study can be found in online repositories. The names of the repository/repositories and accession number(s) can be found in the article/supplementary material.

## Ethics statement

The animal study was reviewed and approved by Institutional Animal Care Committee, Northeast Agricultural University (Harbin, China).

## Author contributions

MZ and YoZ designed the experiments. MZ, HB, YuZ, RW, GL, and PJ conducted the experiments. MZ and HB analyzed the data. MZ, YoZ, and PJ wrote the manuscript. All authors approved the final manuscript.

## Funding

This study was financially supported by China Agriculture Research System of MOF and MARA.

## Conflict of interest

The authors declare that the research was conducted in the absence of any commercial or financial relationships that could be construed as a potential conflict of interest.

## Publisher's note

All claims expressed in this article are solely those of the authors and do not necessarily represent those of their affiliated organizations, or those of the publisher, the editors and the reviewers. Any product that may be evaluated in this article, or claim that may be made by its manufacturer, is not guaranteed or endorsed by the publisher.

## Supplementary material

Supplementary Figure 1 ASV VennDiagram across treatments.

Supplementary Figure 2 Rarefaction curves of the operational taxonomic units (ASV).

Supplementary Table 1 Summary of sequencing data and average length.

Supplementary Table 2 Relative abundances at the phylum level across samples.

Supplementary Table 3 Relative abundances at the genus level across samples.
