# Supplementary figures and images for: Effects of supplementation with lysophospholipids on performance, nutrient digestibility, and bacterial communities of beef cattle
Source: Front Vet Sci. 2022 Jul 22;9:927369. doi: 10.3389/fvets.2022.927369 (PMC9356077; doi:10.3389/fvets.2022.927369)

**Figure S1.** ASV VennDiagram across treatments.

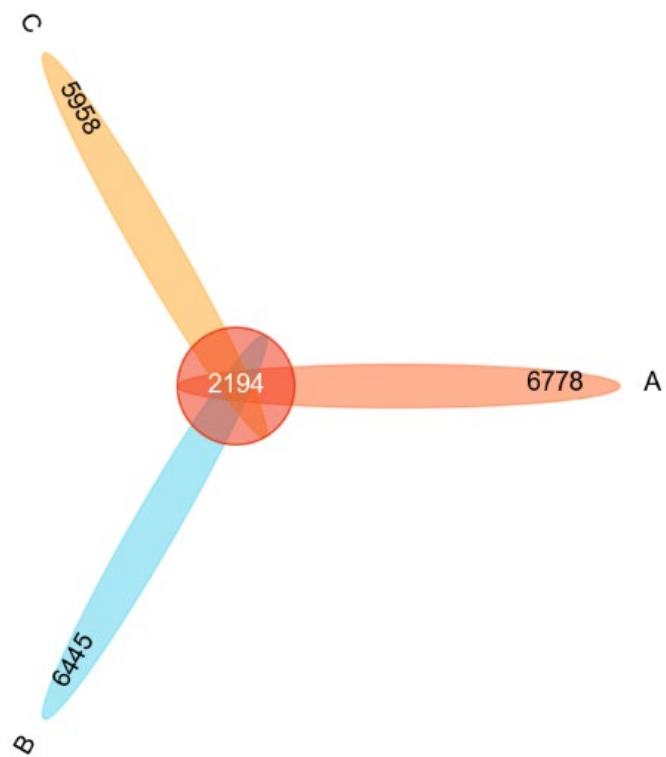

Supplement: Supplementary Figure 1 — ASV VennDiagram across treatments. [file Image_1.pdf]

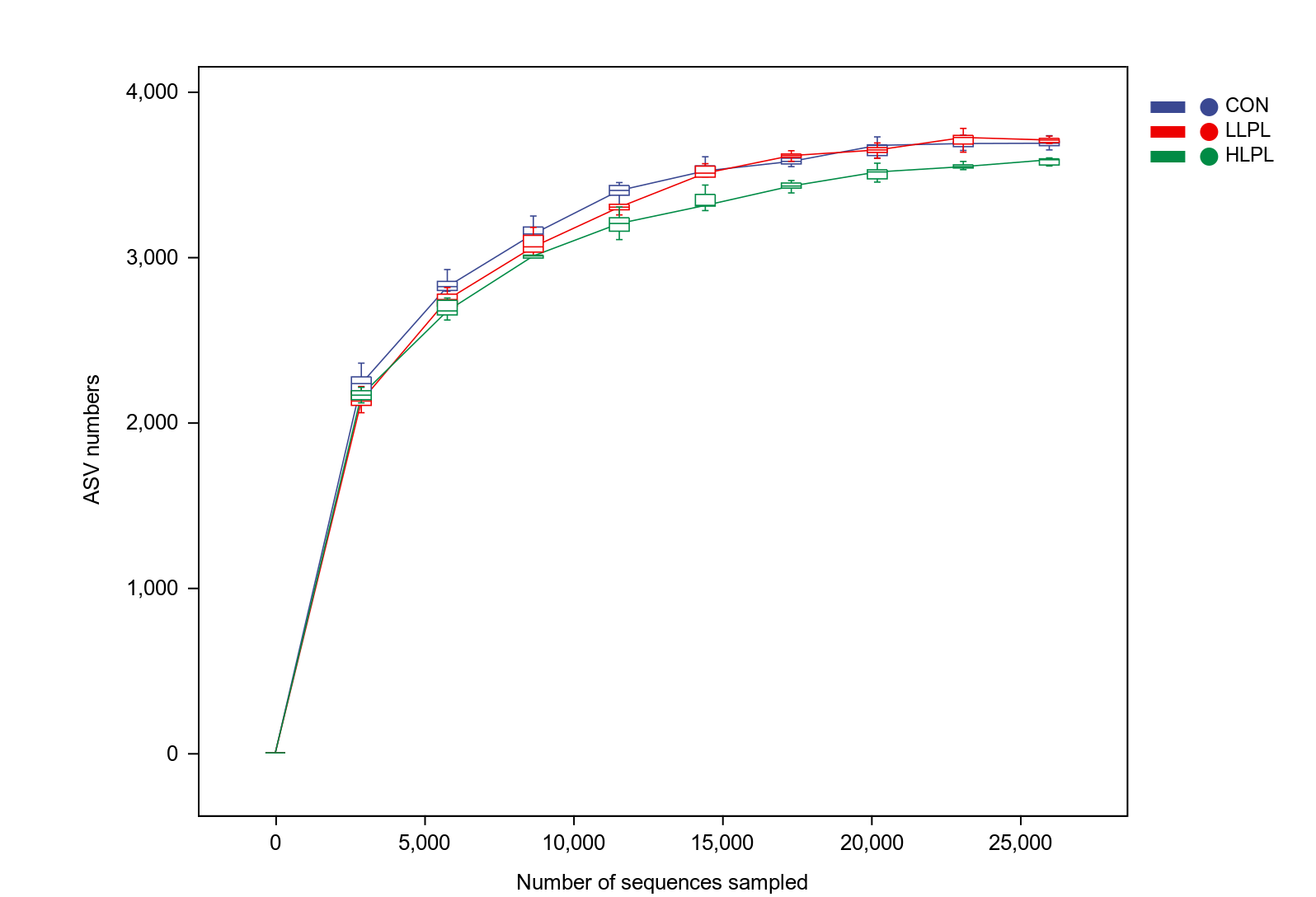

Supplement: Supplementary Figure 2 — Rarefaction curves of the operational taxonomic units (ASV). [file Image_2.png]
