# Supplementary material for: Effects of supplementation with lysophospholipids on performance, nutrient digestibility, and bacterial communities of beef cattle
Source: Front Vet Sci. 2022 Jul 22;9:927369. doi: 10.3389/fvets.2022.927369 (PMC9356077; doi:10.3389/fvets.2022.927369)
Supplement: Supplementary Table 1 — Summary of sequencing data and average length. [file Table_1.DOCX]

**Table S1.** Summary of sequencing data and average length.

| SampleID | Input | Filtered | Denoised | Merged | Nonchimeric | Nonsingleton |
| --- | --- | --- | --- | --- | --- | --- |
| A1 | 66203 | 59745 | 56055 | 39861 | 31948 | 29953 |
| A2 | 105554 | 96512 | 91965 | 67844 | 55235 | 52668 |
| A3 | 106288 | 97163 | 92753 | 73472 | 64605 | 63218 |
| A4 | 84688 | 75945 | 71783 | 53481 | 45585 | 43888 |
| A5 | 70865 | 64542 | 60516 | 43735 | 36828 | 35657 |
| B1 | 105976 | 96682 | 92058 | 70921 | 59290 | 57475 |
| B2 | 89102 | 80941 | 77109 | 58640 | 45624 | 43359 |
| B3 | 89088 | 81085 | 77932 | 63316 | 55604 | 54657 |
| B4 | 94194 | 85838 | 82193 | 63351 | 52234 | 50448 |
| B5 | 75456 | 68490 | 64835 | 48051 | 39691 | 38330 |
| C1 | 105039 | 96384 | 93268 | 82386 | 76321 | 75681 |
| C2 | 61785 | 55778 | 52203 | 34426 | 28690 | 27352 |
| C3 | 91988 | 84291 | 80364 | 59766 | 50222 | 48699 |
| C4 | 100157 | 92217 | 88173 | 67956 | 57715 | 56384 |
| C5 | 99957 | 92314 | 88230 | 65574 | 53133 | 51192 |

| length | count |
| --- | --- |
| 16 | 103 |
| 49 | 18 |
| 50 | 601 |
| 51 | 85 |
| 191 | 2 |
| 209 | 2 |
| 229 | 6 |
| 240 | 2 |
| 261 | 2 |
| 275 | 2 |
| 292 | 2 |
| 293 | 2 |
| 300 | 3 |
| 306 | 2 |
| 318 | 7 |
| 320 | 7 |
| 323 | 6 |
| 325 | 2 |
| 326 | 2 |
| 327 | 2 |
| 328 | 3 |
| 336 | 2 |
| 337 | 36 |
| 338 | 14 |
| 341 | 4 |
| 342 | 3 |
| 348 | 2 |
| 349 | 2 |
| 355 | 2 |
| 359 | 2 |
| 360 | 2 |
| 362 | 2 |
| 365 | 2 |
| 370 | 2 |
| 371 | 7 |
| 374 | 2 |
| 378 | 2 |
| 379 | 2 |
| 382 | 2 |
| 384 | 2 |
| 393 | 2 |
| 394 | 3 |
| 397 | 2 |
| 398 | 2 |
| 404 | 112857 |
| 405 | 106554 |
| 406 | 58073 |
| 407 | 63789 |
| 408 | 147774 |
| 409 | 11614 |
| 410 | 739 |
| 411 | 1532 |
| 412 | 40 |
| 413 | 2411 |
| 414 | 559 |
| 415 | 309 |
| 416 | 71 |
| 417 | 18 |
| 418 | 30 |
| 419 | 1698 |
| 420 | 670 |
| 421 | 174 |
| 422 | 2490 |
| 423 | 7275 |
| 424 | 113120 |
| 425 | 75417 |
| 426 | 2145 |
| 427 | 347 |
| 428 | 58 |
| 429 | 2021 |
| 430 | 36172 |
| 431 | 3603 |
| 432 | 203 |
